# Electrical Properties of Midwave and Longwave InAs/GaSb Superlattices Grown on GaAs Substrates by Molecular Beam Epitaxy

**DOI:** 10.1186/s11671-018-2612-4

**Published:** 2018-07-05

**Authors:** D. Benyahia, Ł. Kubiszyn, K. Michalczewski, J. Boguski, A. Kębłowski, P. Martyniuk, J. Piotrowski, A. Rogalski

**Affiliations:** 1 Institute of Applied Physics, Military University of Technology, 2 Witold Urbanowicz Str., 00-908 Warsaw, Poland; 2 Vigo System S.A., 129/133 Poznańska Str., 05-850 Ożarów Mazowiecki, Poland

**Keywords:** Molecular beam epitaxy, Type-II superlattices, Hall effect, High-resolution X-ray diffraction

## Abstract

In the present work, we report on the in-plane electrical transport properties of midwave (MWIR) and longwave infrared (LWIR) InAs/GaSb type-II superlattices (T2SLs) grown by molecular beam epitaxy (MBE) system on GaAs (001) substrate. The huge lattice mismatch between the T2SL and GaAs substrate is reduced by the growth of GaSb buffer layer based on interfacial misfit array (IMF) technique. In order to compensate the strain in the InAs/GaSb T2SL, we utilized a special shutters sequence to get InSb-like and GaAs-like interfaces. It is found that the MWIR InAs/GaSb T2SL exhibits a *p*- and *n*-type conduction at low and high temperatures, respectively. Interestingly, the conduction change temperature is observed to be dependent on the growth temperature. On the other hand, LWIR T2SL conduction is dominated only by electrons. It is important to note that the dominant scattering mechanism in LWIR T2SL at low temperatures is the interface roughness scattering mechanism.

## Background

Since InAs/GaSb T2SL has been conceptualized by Sai-Halasz et al. [1] in 1977, great attentions have been paid in the investigation of this semiconductor material. Photodetectors based on this T2SL present theoretically higher potential over mercury cadmium telluride (HgCdTe) and the state-of-the-art infrared material systems for the next generation of infrared (IR) applications [2, 3]. Interestingly, InAs/GaSb T2SL exhibits an unusual type-II broken gap band lineup where the InAs conduction band minimum is located 140 meV lower than the GaSb valence band top [1]. Consequently, the fundamental transition between the heavy-hole subbands and the conduction band bottom depends on the thickness of the InAs or GaSb layer [4]. However, the main advantage of this alignment is the reduction of Auger recombination rate thanks to the suppression of some non-radiative pathways in the valence band [5]. In addition, the band-to-band tunneling is decreased significantly due to the large effective masses (≈ 0.04 *m*_0_) of electrons and holes [6]. These two latter features permit the reduction of the dark current, which leads to the high operation temperature (HOT) of the photodetector.

InAs/GaSb T2SL is traditionally grown on lattice-matched GaSb substrate. However, this latter is expensive and available in small sizes less than 3 in., which impede the realization of large-format focal plane arrays (FPAs). Moreover, GaSb substrates are not “epi-ready” and their growth surfaces contain many macroscopic defects [7]. Furthermore, the absorption coefficient is relatively high in GaSb substrate (≈ 100 cm^−1^) for IR radiation above 5 μm [8]. Due to its numerous advantages, GaAs has been proposed as a viable candidate for the growth of InAs/GaSb T2SL [9–12]. Indeed, they are “epi-ready,” cost-efficient, and available in large sizes up to 6 in. Besides, GaAs has an absorption coefficient two orders of magnitude lower than that of GaSb. Unfortunately, a huge lattice mismatch (~ 7.5%) exists between GaAs and InAs/GaSb T2SL that results in high misfit dislocation density (10^9^ cm^−2^) [13]. Therefore, it is compulsory to concept new growth techniques to relieve the strain and reduce the dislocation density. Among these techniques are low-temperature nucleation [14] and IMF technique [15, 16].

In order to improve the performances of photodetectors based on InAs/GaSb T2SL, a better understanding of fundamental parameters is needed. One of these parameters is the background carrier concentration which is associated with the minority carrier lifetime and diffusion lengths. It is worth noting that InAs and GaSb bulk materials have opposite polarity of carriers’ concentration. Indeed, InAs and GaSb materials grown using molecular beam epitaxy (MBE) are residually *n*- and *p*-type, respectively [17, 18]. Consequently, the conduction of the InAs/GaSb T2SL is predicted to be dependent on the thickness of each constituent.

In this paper, we investigate the in-plane transport properties of 10 ML InAs/10 ML GaSb and 24 ML InAs/7 ML GaSb T2SLs dedicated for the detection in MWIR and LWIR regions, respectively, grown on semi-insulating GaAs (001) substrates. This study is achieved by performing a temperature-dependent Hall effect measurement using the Van der Pauw method. Besides, the influence of the growth temperature on the conduction of the InAs/GaSb T2SL is presented.

## Methods

InAs/GaSb T2SL samples have been grown on semi-insulating GaAs (001) substrates in a RIBER Compact 21-DZ solid source MBE system. This latter is equipped with standard effusion cells for group III elements (indium (In) and gallium (Ga)) and valved cracked cells for group V materials (arsenic (As) and antimony (Sb)). The cracker temperatures were kept at 900 °C for both As and Sb to produce As_2_ and Sb_2_, respectively. The manipulator thermocouple (TC) and BandiT (BT) are utilized to monitor the growth temperature. This latter has been calibrated from the GaAs oxide desorption temperature. Following the deoxidization of GaAs substrates at 610 °C (measured by BT), a 250-nm-thick GaAs layer was deposited at 585 °C (BT) to get a smooth starting surface. Subsequently, a 1-μm-thick GaSb buffer layer has been grown using IMF technique at a BT temperature of 440 °C [16, 19]. This technique consists on the formation of a periodic array of 90° misfit dislocation at the GaAs/GaSb interface leading to a low dislocation density (≈ 10^6^ cm^−2^) [20]. After the growth of GaSb buffer layer, the BT cannot be used anymore due to the emissivity changes, surface roughening, and extra radiative absorption mechanisms [21]. Thus, the growth temperature of the InAs/GaSb T2SL is controlled only by the TC. MWIR 10 ML InAs/10 ML GaSb T2SLs are grown at different substrate temperatures, 330, 390, and 400 °C (TC) to investigate the influence of the growth temperature on the transport properties. On the other hand, LWIR 24 ML InAs/7 ML GaSb T2SL has been deposited at only 390 °C. In order to compensate the strain between InAs and GaSb, special shutters sequence, which was reported to lead to a better structural quality [22, 23], was used as follows: growth of InAs was followed by Sb soak of 8 s to form InSb-like bonds, whereas GaSb growth was followed by 2 s of As soak to grow GaAs-like interface. The V/III flux ratio is 8.3 and 4.6 for InAs and GaSb, respectively. Besides, the growth rate is 0.5 ML/s for both InAs and GaSb. The growth was monitored in situ by reflection high-energy electron diffraction (RHEED) system.

The grown samples have been assessed by high-resolution X-ray diffraction (HRXRD) of PANalytical X’Pert to investigate the structural properties. The Cu Kα_1_ radiation (*λ* ≈ 1.5406 Å) originating from a line focus and a four bounce Ge (004) monochromator have been utilized. The transport properties were evaluated by Hall effect measurements using the Van der Pauw method in an ECOPIA system, with a temperature range of 80–300 K. Measurements were performed on square samples of 6 × 6 mm^2^; contact was made by indium dots in each corner. A magnetic field of 0.4 T was applied normal to the samples.

## Results and Discussion

**Fig. 1:**
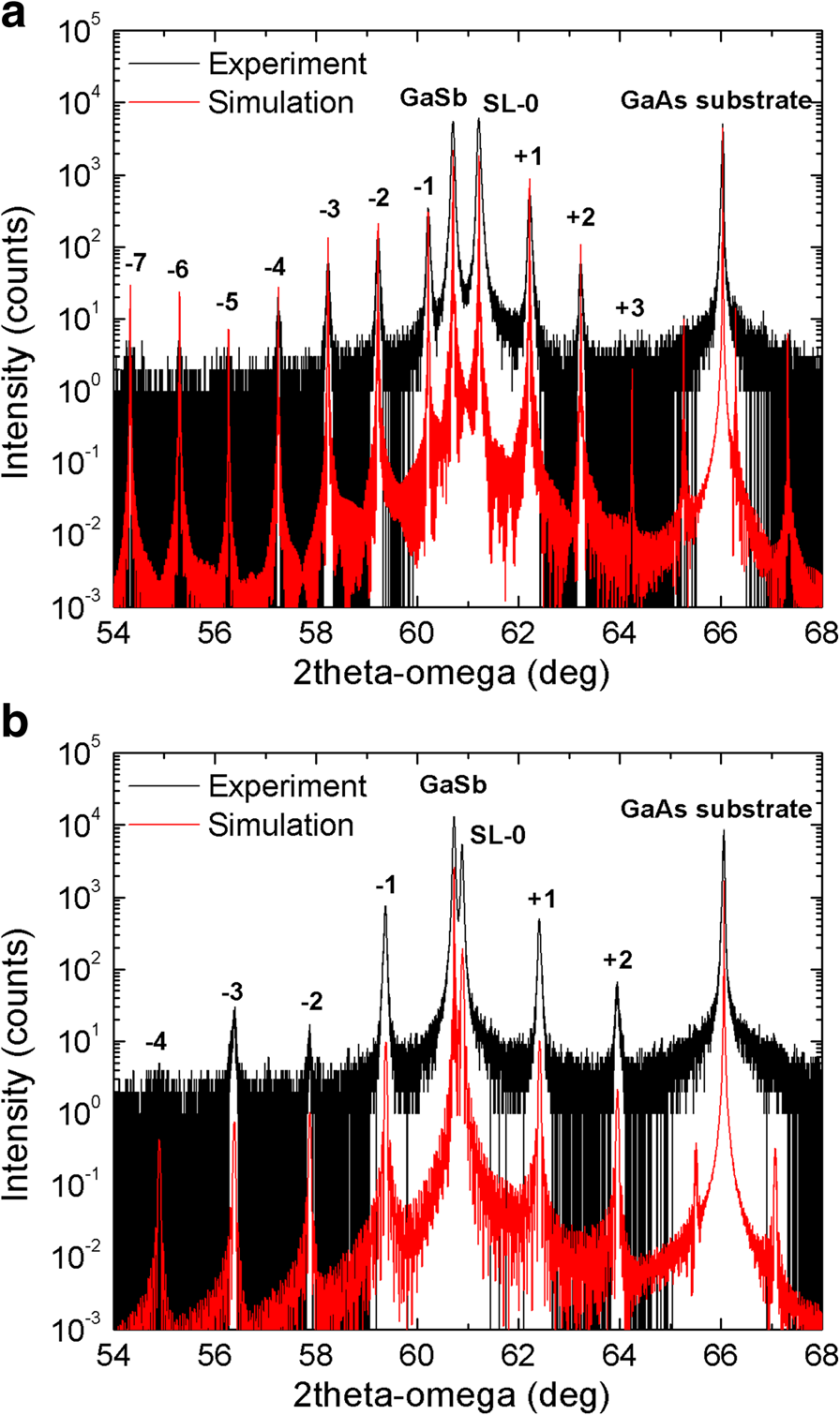
HRXRD 004 *2θ-ω* scan of **a** MWIR **b** and LWIR InAs/GaSb T2SLs. Experimental (black line) and simulated (red line) HRXRD *2θ-ω* scans for the (004) reflection of **a** MWIR T2SL; there are well-resolved satellites with an order up to 4, which is a characteristic of good quality of superlattice. The FWHM of the zeroth-order peak is 107 arcsec, **b** and LWIR InAs/GaSb T2SLs; there are satellite peaks with an order up to 7, which confirms the high crystalline quality. The FWHM of the zeroth-order peak is 99 arcsec. The period of each superlattice is calculated from the distance between adjacent satellites

**Fig. 2:**
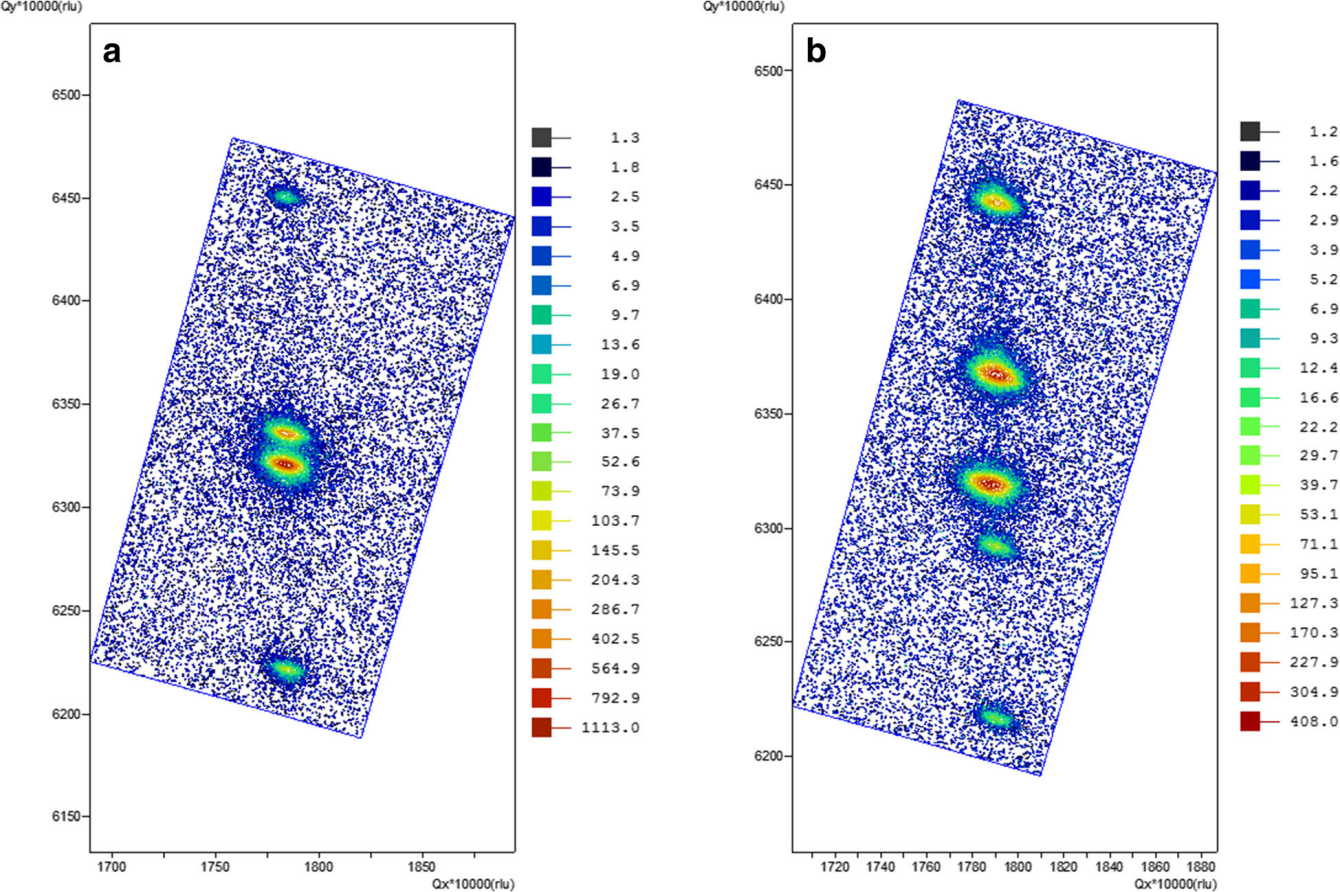
Asymmetric 115 RSM of **a** MWIR and **b** LWIR InAs/GaSb T2SLs. The reciprocal space map for the asymmetric reflection (115) of **a** MWIR and **b** LWIR InAs/GaSb T2SLs. The peaks in both superlattices are aligned vertically (they have the same value of the scattering vector Q_x_). Therefore, the two superlattices for MWIR and LWIR are practically fully strained

Figure 1 illustrates the measured and simulated HRXRD *2θ-ω* scanning curves of the symmetric (004) reflection for the MWIR and LWIR InAs/GaSb T2SLs. The simulation is performed by the “Epitaxy” software provided by PANalytical X’Pert. As can be seen, there are well-resolved satellites with an order up to 4 for MWIR T2SL and up to 7 for LWIR one. This indicates the high structural quality of the grown layers, especially for LWIR T2SL. On the other hand, the full width at half maximum (FWHM) of the zeroth-order peak measured in *ω-2θ* direction is 107 and 99 arcsec for MWIR and LWIR T2SLs, respectively. The superlattice period (*L*) is determined from the angle distance between two adjacent satellites (Δ*θ*) as follows:

1 $$ L=\lambda /\left(2\times \Delta  \theta \times \mathit{\cos}{\theta}_{SL}\ \right) $$

where *λ* is the wavelength of the incident X-ray beam mentioned previously and *θ*_*SL*_ is the Bragg angle of the zeroth-order peak of the superlattice. From Fig. 1, the period of the MWIR and LWIR T2SLs is 6.74 ± 0.01 and 10.24 ± 0.02 nm, respectively. By fitting the measured curve with the simulated one, the composition of one period of MWIR T2SL is found to be as follows: GaSb 3.4 nm (11.2 ML), GaAs 0.1 nm (0.2 ML), InAs 3.0 nm (10.1 ML), and InSb 0.2 nm (0.5 ML). Moreover, the thicknesses of LWIR T2SL constituents are as follows: GaSb 2.3 nm (7.5 ML), GaAs 0.1 nm (0.2 ML), InAs 7.4 nm (24.7 ML), and InSb 0.4 nm (1 ML). The lattice mismatch determined from the angle between the zeroth-order peak and the GaSb buffer layer is 8.9 × 10^−3^ and 4.5 × 10^−3^ for MWIR and LWIR T2SLs, respectively. Figure 2 presents the asymmetric (115) reciprocal space map (RSM) for the grown T2SLs. In both samples, the satellites of the superlattices and the GaSb peak are aligned vertically (they have the same component of the scattering vector Q_x_), which leads to the conclusion that both T2SLs are practically fully strained.

**Fig. 3:**
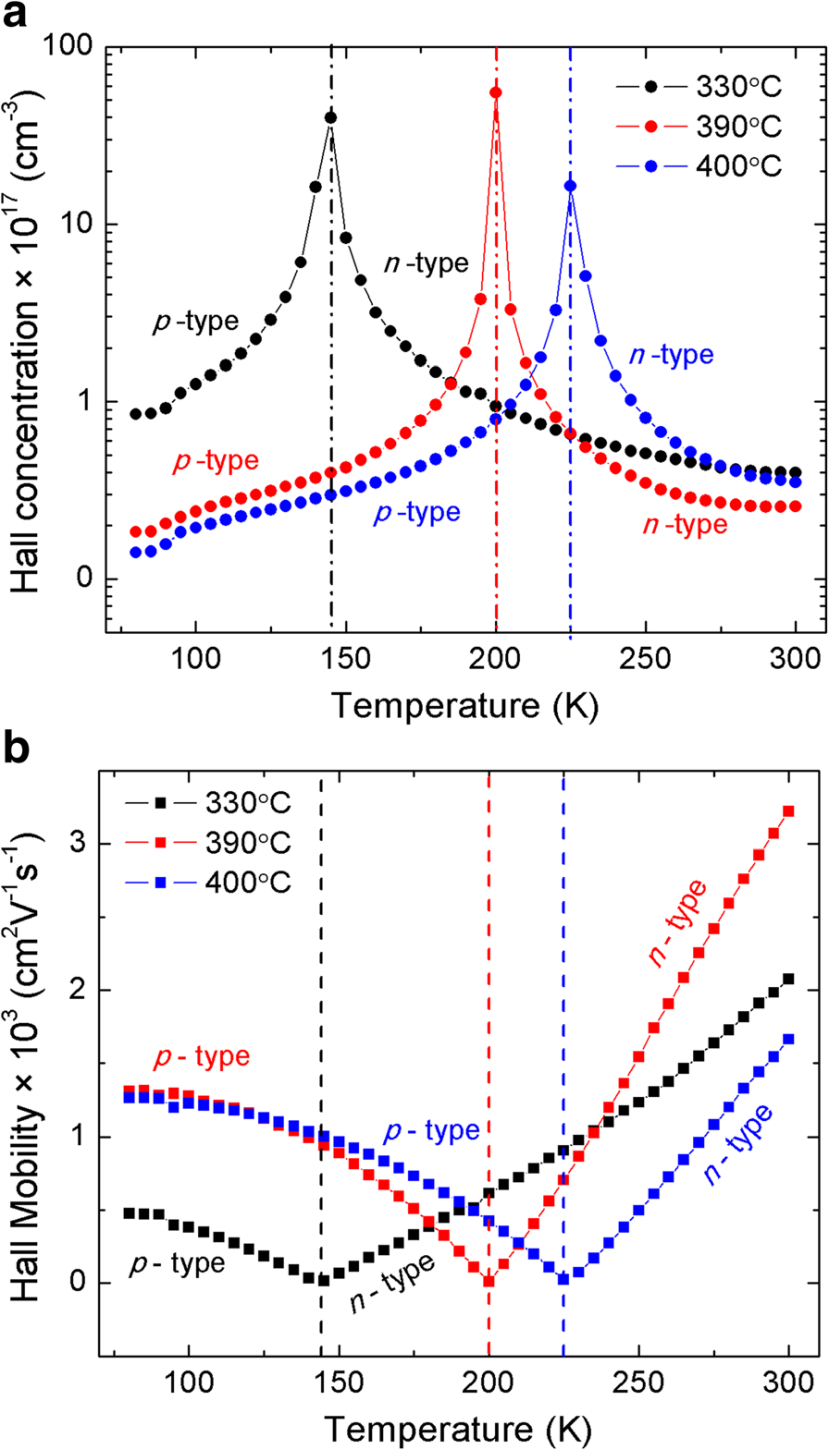
**a** Hall concentration and **b** Hall mobility of MWIR InAs/GaSb T2SL grown at different temperatures. The electrical parameters of the MWIR InAs/GaSb T2SLs grown at different temperatures. **a** Hall concentration: the three T2SLs exhibit a change of conductivity. They are *p*-type at low temperatures and *n*-type at high temperatures. **b** Hall mobility: there are two regions for the mobility tendency. For low temperature, the mobility decreases due to the different scattering mechanisms. For high temperatures, the mobility increases by increasing the temperature, which can be explained by the ionized traps in InSb-like interface. The temperature at which the conductivity change occurs increases when the growth temperature increases, which is due to the high defect levels at higher temperatures

The in-plane electrical parameters of the grown MWIR InAs/GaSb T2SLs are illustrated in Fig. 3. As it can be seen, the unintentionally doped InAs/GaSb T2SL exhibits a reproducible change in type of conductivity. In spite of the influence of GaSb buffer layer (*p*-type) on the Hall effect measurements, it should be noted that the change in the conductivity type is due only to the T2SL layer. This change was also reported by several groups [6, 24–26]. The T2SL exhibits a *p*-type conduction below the temperature at which the change occurs (*T*_*ch*_) and an *n*-type conduction above *T*_*ch*_. As mentioned previously, the InAs and GaSb layers exhibit an *n*-type and *p*-type conduction, respectively. Therefore, the residual background of the InAs/GaSb T2SL with similar thickness for the two constituents is induced by the majority carrier compensation from the binary constituents of the T2SL [27]. For the value of *T*_*ch*_, Mohseni et al. [6] reported a value of 140 K, Christol et al. [24] got a value of 190 K, while Khoshakhlagh et al. [25] pointed out a value of 200 K. The behavior of the sheet carrier concentration and mobility is governed by the well-known intrinsic phonon scattering (acoustic, piezoelectric, polar, and nonpolar optical) mechanisms. Exceptionally, the Hall mobility increases with increase of the temperature above *T*_*ch*_ (Fig. 3b); this is *probably* because of the ionized traps due to the InSb interface at the GaSb-on-InAs interface [6]. *T*_*ch*_ value is 145, 195, and 225 K, for the T2SL grown at 330, 390, and 400 °C, respectively (Fig. 3); this is due *probably* to the high hole concentration at higher growth temperature, which shifts the *T*_*ch*_ to lower temperature. The high hole concentration is due to defects and ionized vacancies at high growth temperature. The InAs/GaSb T2SL grown at 390 °C is characterized by a carrier’s concentration of 1.8 × 10^16^ and 2.5 × 10^16^ cm^−3^ at 80 and 300 K, respectively. This result is better than that reported by Mohseni et al. [6] (Hall concentration ranges from 1.5 to 4 × 10^17^ cm^−3^) and practically the same as that reported by Christol et al. [24] (Hall concentration of 1.6 × 10^16^ and 6 × 10^16^ cm^−3^ at 100 and 300 K, respectively). On the other hand, the Hall mobility is 1300 (*p*-type) and 3200 cm^2^/V s (*n*-type), at 80 and 300 K, respectively. The reached mobility is much higher than that reported by Christol et al. [24], who got a Hall mobility of 100 and 1800 cm^2^/V s at 100 and 300 K, respectively.

**Fig. 4:**
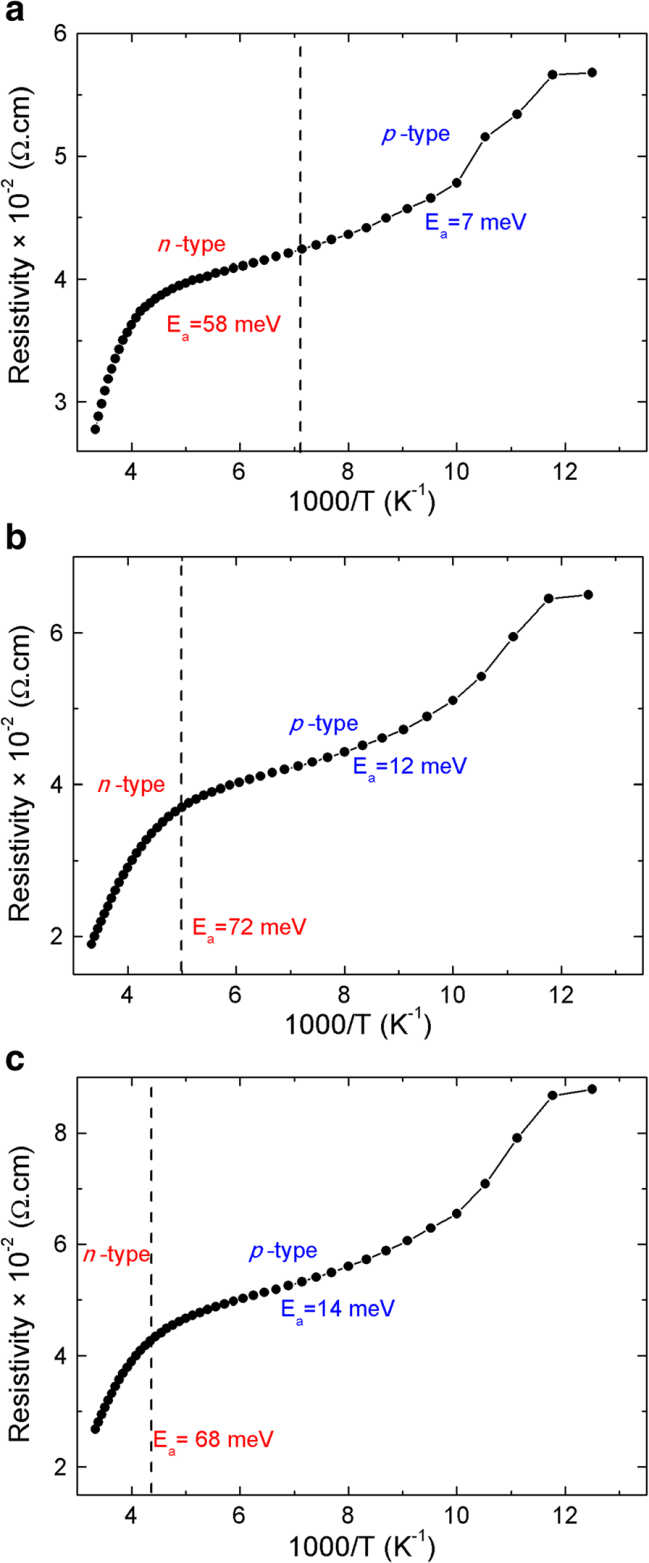
Hall resistivity of the MWIR InAs/GaSb T2SL grown at **a** 330 °C, **b** 390 °C, and **c** 400 °C. The Hall resistivity of the MWIR InAs/GaSb T2SL deposited at different growth temperatures, **a** 330 °C, **b** 390 °C, and **c** 400 °C. For each graph, there are two well-defined slopes. From the Arrhenius law, two thermal energies can be extracted, which confirms the existence of two impurity levels. One represents the *n*-type carriers, and the second one corresponds to the *p*-type carriers. The deep impurity level associated to the *n*-type carriers is the result of the band lineup between InAs and InAs/GaSb T2SL

The resistivity of the three different samples is shown in Fig. 4. It can be noticed that the resistivity and temperature have two well-defined slopes. For each sample, two thermal activation energies can be extracted from the Arrhenius law. For the *n*-type region, the activation energy *E*_*an*_ is 58, 72, and 68 meV for the T2SL grown at 330, 390, and 400 °C, respectively. While for the *p*-type region, *E*_*ap*_ is equal to 7, 12, and 14 meV, for the InAs/GaSb T2SL deposited at 330, 390, and 400 °C, respectively. For low temperatures (below *T*_*ch*_), the T2SL shows a *p*-type due to *p*-type carriers associated with *E*_*ap*_ which dominate the carrier generation and recombination mechanisms. For temperatures above *T*_*ch*_, the T2SL exhibits an *n*-type conduction due to the activation of deep-level carriers associated with high activation energy *E*_*an*_. The source of these deep levels is the shallow levels in the bulk InAs that is the result of the band lineup between InAs and InAs/GaSb T2SL and which acts as deep levels in the InAs/GaSb T2SL [28].

**Fig. 5:**
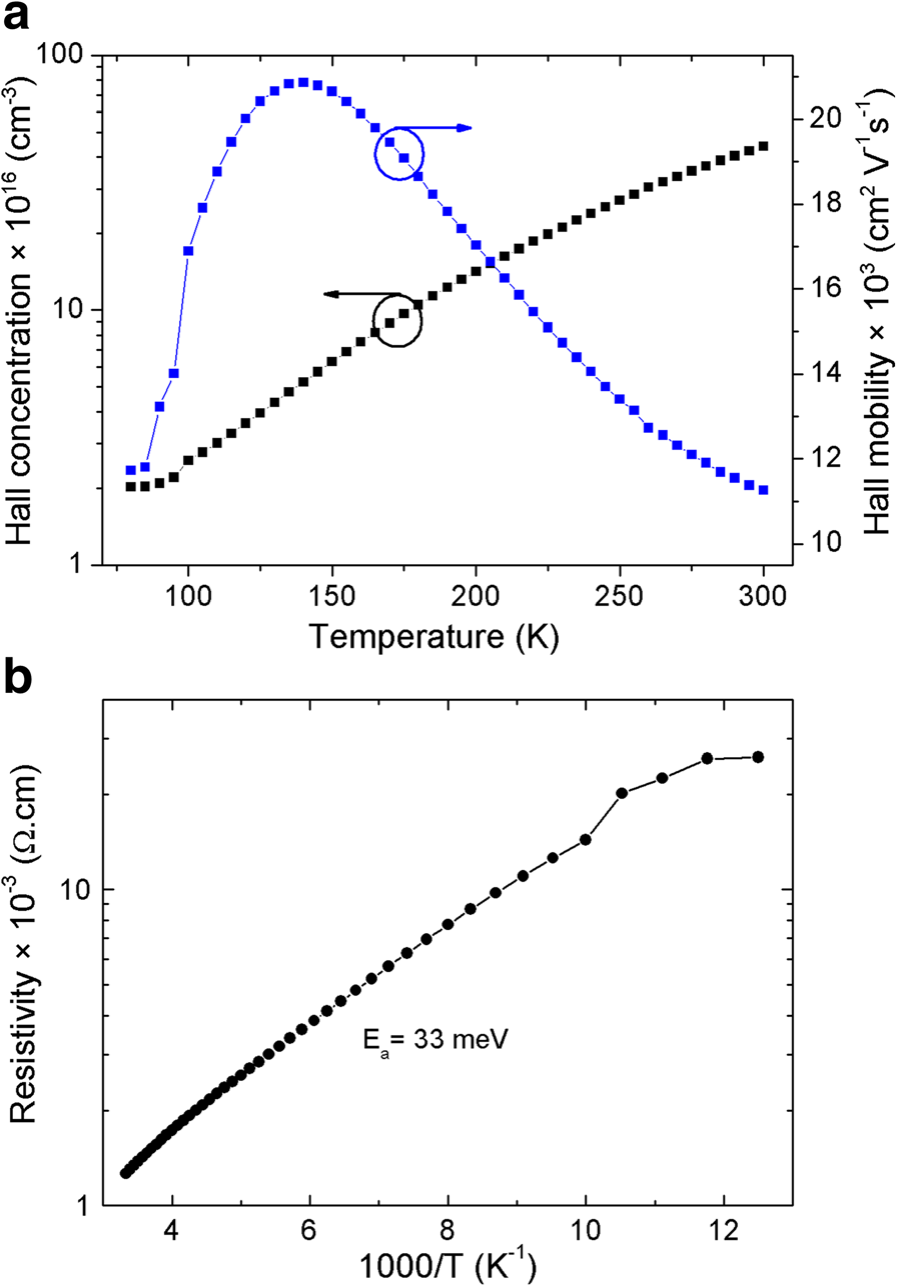
**a** Hall concentration, Hall mobility, and **b** Hall resistivity of unintentionally doped LWIR InAs/GaSb T2SL. The transport properties of the LWIR InAs/GaSb T2SL. **a** Hall concentration and mobility: this superlattice exhibits only *n*-type conduction for the whole temperature range. The Hall concentration increases by increasing the temperature which is caused by the ionization effects. On the other hand, the Hall mobility behavior is governed by the different scattering mechanisms (phonons and ionized impurities) for temperatures higher than 95 K. For temperatures below 95 K, the mobility is constant (as well as the Hall concentration), which confirms the existence of another scattering mechanism which is temperature-independent. This latter is the interface roughness mechanism. **b** Hall resistivity: from the Arrhenius law, there is only one thermal energy associated to one impurity level

The Hall concentration, mobility, and resistivity of the LWIR InAs/GaSb T2SL are presented in Fig. 5. Opposite to the MWIR T2SL, there is no change of type of conductivity in the case of this sample. This T2SL exhibits an *n*-type conduction. The influence of the *p*-type GaSb buffer (low-mobility carriers) layer on the Hall effect measurement for this *n*-type T2SL (high-mobility carriers) is negligible, since the Hall mobility is proportional to the carriers’ mobility squared. Khoshakhlagh et al. [25] reported the same result for 13 ML InAs/7 ML GaSb T2SL. In addition, Szmulowicz et al. [29] pointed out that LWIR T2SL, in which the InAs layer is thicker than GaSb, tends to be *n*-type. This *n*-type conduction is due to the large thickness of InAs layer (residually *n*-doped.) compared to that of GaSb. These *n*-type carriers are associated with an activation energy of 33 meV (Fig. 5b). The behavior of the Hall concentration and mobility is typical for *n*-type semiconductors, except the temperature range below 95 K, where the carrier’s concentration and mobility are almost temperature-independent. This is an indication of the existence of a temperature-independent scattering mechanism in this temperature region. This latter is demonstrated to be the interface roughness scattering (IRS) mechanism [30–34]. This mechanism is dominant at low temperature, where the phonon scattering is frozen out [35]. IRS mechanism is due to the existence of interfaces, as well as the variation of the layers’ thicknesses, which results in the variation of the electronic energy levels; therefore, they act as a source of carrier scattering [35]. Moreover, the Hall mobility dominated by the IRS mechanism is proportional to the sixth power of the InAs thickness ($\mu \propto {d}_{\mathrm{InAs}}^{6.2}$) [35].

## Conclusions

In summary, InAs/GaSb T2SLs have been grown on GaAs substrate using GaSb buffer layer based on IMF technique. Moreover, these T2SLs have been demonstrated for MWIR and LWIR detection regions. It has been found that MWIR T2SL exhibits a change in the conduction type, form *p*- to *n*-type as the temperature increases. Furthermore, the temperature at which the change occurs increases as the growth temperature of the T2SLs increases. This conduction type change is attributed to the existence of two impurity levels with two different activation energies. On the other hand, the LWIR InAs/GaSb T2SL conduction is demonstrated to be *n*-type for the whole range of temperature. In addition to the conventional scattering mechanisms, the IRS mechanism is proved to be the dominant scattering mechanism at low temperatures. These results allow a better understanding of the physical properties of the InAs/GaSb T2SL, which leads to the improvement of IR photodetector performances based on this material.

## Abbreviations

BT: BandiT
FPAs: Focal plane arrays
FWHM: Full width at half maximum
HOT: High operation temperature
HRXRD: High-resolution X-ray diffraction
IMF: Interfacial misfit array
IR: Infrared
IRS: Interface roughness scattering
LWIR: Longwave infrared
MBE: Molecular beam epitaxy
MWIR: Midwave infrared
RHEED: Reflection high-energy electron diffraction
RSM: Reciprocal space map
T2SL: Type-II superlattice
TC: Thermocouple
